# Testing learning as alternative to the blank slate hypothesis in the honey bee, *Apis mellifera*

**DOI:** 10.1371/journal.pone.0325591

**Published:** 2025-06-09

**Authors:** Olav Rueppell, Kayla De Jong, Jacob J. Herman, Cleo Randall

**Affiliations:** Department of Biological Sciences, University of Alberta, Edmonton, Alberta, Canada; Albert-Ludwigs-Universitat Freiburg, GERMANY

## Abstract

Reliable recognition of nestmates and discrimination against non-nestmates is key to the integrity of social insect colonies. Cuticular hydrocarbon profiles play a key role in this recognition process in many species, including honey bees. Newly emerged worker bees are largely devoid of cuticular hydrocarbons and therefore believed to represent a “blank slate” that is not discriminated against and instead accepted into other colonies regardless of colony origin. However, instead of being unrecognizable, the absence of cuticular hydrocarbons may also represent a recognizable “Gestalt”. Thus, an alternative hypothesis for the universal acceptance of newly emerged workers may be that older workers in every colony learn the absence of cuticular hydrocarbons as a familiar stimulus that belongs to their colony because other such workers are constantly emerging under normal circumstances. Here, we tested this hypothesis by comparing the response to newly emerged workers between bees that matured in colonies with and without newly emerging bees. Contrary to our prediction, we found no significant difference between these two experimental groups in an aggression bioassay towards newly emerged workers. We thus failed to provide empirical evidence against the blank slate hypothesis. However, the groups displayed significant differences in aggression towards foragers from their own respective colonies, indicating that the emergence of new workers in a colony can affect group discriminatory behavior in honey bees. Furthermore, we identified a negative effect of temperature on aggressive behavior toward newly emerged workers.

## Introduction

Many social insect species live in coordinated and complex societies [1]. These colonies function due to the cooperation among its members and rely on the exclusion of non-members [2]. Social insect colonies typically occupy discrete nests that can be defended, used for food storage, and serve as a protected environment for colony members [3]. Thus, vulnerable colony members and exploitable resources need to be protected from hetero- or conspecific parasites and predators by excluding non-nestmates from the nest. Such exclusion requires the distinction of nestmates from alien individuals, a process called nestmate recognition.

Nestmate recognition has been studied in detail in the socially complex western honey bee (*Apis mellifera*). Similar to other social insects, honey bees face conspecific pressure in form of honey robbing, particularly when food is scarce [4]. Nestmate recognition is particularly critical at the nest entrance and specialized guard bees detect invaders, triggering an aggressive biting, dragging, or stinging response [5]. In the behavioral ontogeny of workers, guarding behavior is a transitory period between in-hive tasks and outside foraging and it is only performed by a small proportion of workers [6]. Despite the small number of guard bees at the entrance detecting and attacking invaders, the vast majority of workers is presumably capable of nestmate recognition and discrimination against non-nestmates occurs also inside the bee hive [7] although some context-dependency exists [8].

The mechanisms of nestmate recognition are still not completely understood but the olfactory profile of individuals, specifically the cuticular hydrocarbons (CHCs), play a key role [2]. This profile is a multi-variate chemical blend and nestmate recognition relies on comparing individual profiles with a “Gestalt” template that reflects a bee’s representation of its own colony [9]. The origin of the individual- and colony-level CHC profiles is complex and influenced by individual, microbiome, and colony environmental factors [8,10–12]. While learning has been established as an important component of nestmate recognition [13–15], it is also not yet clear what specific aspects of the profiles are determining the outcome of an assessment [12,16].

CHCs also serve non-communicative functions and correlate with behavioral status of honey bee workers [8,17]. Young, newly emerged honey bees are largely devoid of distinct cuticular hydrocarbons [8,18] and are readily accepted by other colonies. These concurrent observations may be due to a lack of discriminatory cues eliciting no rejection response [19], a concept that was formalized as the “blank slate” hypothesis [18]. Implicit in the blank slate hypothesis and its empirical support in honey bees is the importance of the inability of workers to discriminate against newly emerged bees in the absence of recognition cues [18]. However, this previous test of the blank slate hypothesis did not consider alternative explanations. One such alternative to explain the previous observations could be a learning process [20]: Older bees may tolerate newly emerged bees because they are constantly exposed to newly emerging bees in their colony. Thus, they learn to incorporate the depauperate profile of newly emerged bees into the colony “Gestalt” instead of simply lacking the discriminatory abilities to recognize and discriminate against the absence of cuticular hydrocarbons, which is an important proximal distinction. In this study, we set out to test this new “Learning the Blank Slate” hypothesis as alternative to the original blank slate hypothesis by comparing aggressive behavior towards newly emerged bees by workers that were constantly exposed to newly emerged workers and others that lacked familiarity with newly emerged workers.

## Materials and methods

From July 19^th^ to Aug 2^nd^ 2022, six nuc hives were set up with a queen, approximately 5000 worker bees of mixed ages, and five frames containing honey, pollen, brood, and empty cells in the research apiary of the University of Alberta. Three existing colonies were split into pairs and a new queen was introduced into each half: One colony half in each pair did not receive any capped brood and all developing capped brood was regularly removed throughout the experiment to prevent any new workers from emerging. This group constituted our “no exposure to newly emerged workers” treatment. To keep population sizes in the remaining three “control” treatment colonies comparable, random workers were removed from these colonies as needed. Experimental pairs (3 replicate experiments) were set up one week apart from each other.

One day after colony establishment, the experiment was started with the introduction of color-marked cohorts of 1000 newly emerged bees into each of the experimental colonies. After three weeks of either constant exposure to newly emerging bees (control group) or maturing in a colony without any new emerging bees (treatment group), marked workers from the focal cohorts were transferred into standard Petri dishes. Emergence of newly emerged workers in the control colonies was continual, ensuring that bees in our experimental cohorts were exposed to newly emerged nestmates. Each Petri dish included nine workers and was placed back into the source colony of the workers for 24 hours to provide the workers to acclimatize to this experimental arena in the context of their colony. Each Petri dish top contained a hole in the cover (approximately 3 cm diameter) that was covered by a mesh-screen to allow interactions with the rest of the colony. At the same time, an emerging brood frame was removed from an unrelated colony and kept in an emergence incubator (34 °C and 60% rel. hum.) overnight for bees to emerge and serve as a source of newly emerged workers to be tested as “intruder” in the subsequent aggression assay.

For the actual behavioral test, Petri dishes with the 21–22-day old focal workers were carefully removed from the treatment and control hives and kept on a table for five minutes to acclimatize the bees to the observation conditions. During this period, bees displayed normal in-hive behaviors and showed no signs of alarm or distress. Subsequently, a newly emerged bee (=”intruder”) was carefully introduced into the center of the Petri dish. Subsequent behavioral interactions initiated by the treatment or control bees were classified and recorded for eight minutes. Behavioral interactions were observed and scored as “antennation” (touching intruder with antenna or moving antennae directly towards intruder), “stalking” (moving towards and following intruder for more than 5 sec), “crawl over” (moves directly on top or across intruder), “mandible flaring” (antennation directly towards intruder with open mandibles), “biting” (use of mandibles to grasp intruder), and “stinging” (stinger is visibly moved towards intruder in stinging attempt). Separate instances were scored when performed by different individuals or by the same individual with a minimum of a five-second interruption between two instances.

After removal of the newly emerged bee intruder, the treatment or control bees were given two minutes rest, before a forager from their own respective home colony was introduced and behavioral monitoring was performed as described above. After the removal of this familiar forager, the experimental bees were rested for another two minutes before the procedure was repeated a third time with a forager from an unfamiliar colony. These two additional measures were performed to control for baseline differences in aggression between experimental groups. Experimental time and room temperature were also recorded to control for possible effects on the bees’ behavior [21]. Statistical analyses were performed in “RStudio” using “R” 4.4.3.

An aggregate aggression score for each behavioral assay was calculated by adding the number of all instances of all interactions after multiplying the number of “mandible flares”, “biting”, and “stinging” by two, three, and five respectively, to account for the higher aggression intensity of these behaviors. Three other aggression scores were also calculated (S1 Table) and evaluated. However, the analyses of these alternate scores did not fundamentally change the results and we show here the original score that most clearly differentiated the behavior towards newly emerged bees (NE), same colony foragers (SF), and foreign foragers (FF), as determined by simple ANOVA in “RStudio” (*aov*), followed by Tukey’s post-hoc test (*TukeyHSD*). Following, these NE, SF, and FF scores were each modeled as a function of treatment (fixed effect), temperature (fixed effect covariate), and replicate (random effect) with a generalized linear mixed model using a Tweedie link function [22], implemented in the glmmTMB package (*glmmTMB*). An index for quantifying how much aggressive behavior was specifically directed towards newly emerged workers was calculated by dividing the aggression score against the newly emerged worker in a trial by the sum of the aggression scores against the familiar and the unfamiliar foragers in the same trial. This index score was evaluated with a generalized linear model as described above. Additionally, we evaluated the occurrence of severe aggression (biting and stinging) towards NE, SF, and FF between the treatment groups by Fisher’s exact tests.

## Results

The full range of categories of aggressive behavior was observed ( S1 Table). All four calculated aggression scores were significantly different among the three types of assay performed (behavior toward newly emerged bees, familiar foragers, or unfamiliar foragers) and the score that most clearly differentiated among these groups (F_(2,219)_ = 39.1, p < 0.001) was selected for all subsequent analyses. The bees’ response scores towards unfamiliar foragers was significantly higher than toward newly emerged bees (p < 0.001) and familiar foragers (p < 0.001), while no significant difference was observed between how newly emerged bees and familiar foragers were treated overall (p = 0.691). Across all groups, response scores towards newly emerged bees were not related to the scores towards familiar foragers (r_s_ = −0.13, n = 74, p = 0.257) or unfamiliar foragers (r_s_ = −0.16, n = 74, p = 0.169), while a significant correlation existed among the scores towards familiar and unfamiliar foragers (r_s_ = −0.30, n = 74, p = 0.009).

**Fig 1:**
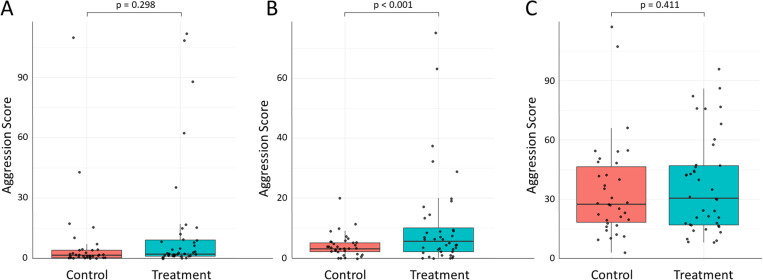
Continuous exposure to newly emerged nestmates affects aggressive behavior of worker honey bees. When comparing the behavior of groups of three-week-old workers that had constant exposure to newly emerged bees (controls) with the behavior of such groups of workers that had no contact with newly emerged workers after their own emergence (treatment), the treatment increased our composite score of aggressive behavior towards foragers from the same colony (B), but not the aggression scores towards newly emerged workers (contrary to our prediction; A) or towards foragers from an unrelated colony (C).

**Fig 2:**
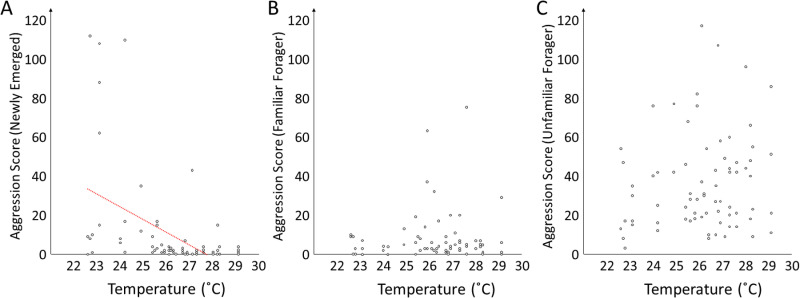
Different temperature effects on aggressive behavior of worker honey bees. Aggression scores towards newly emerged workers were negatively affected by ambient temperature (A). In contrast, the effect of temperature on the same measures towards foragers from the same colony (B) or towards unfamiliar foragers (C) was not significant and positive trends were observed instead.

Aggression scores towards newly emerged workers (n = 74) were significantly influenced by temperature (z = −7.1, p < 0.001) but not by treatment (z = 1.0, p = 0.298; Figs 1 and 2). In contrast, aggression scores towards foragers from the same colony (n = 74) were not significantly influenced by temperature (z = 0.84, p = 0.403) but treatment (z = 4.4, p < 0.001) because workers without exposure to newly emerged bees scored on average 2.6x higher in aggression than the controls (Figs 1 and 2). Aggression scores against unfamiliar foragers were not significantly affected by temperature (z = 1.6, p = 0.102) or treatment (z = −0.82, p = 0.411; Figs 1 and 2).

The occurrence of severe aggression (biting and stinging) was not significantly different between experimental groups towards newly emerged workers (treatment: 10/40, control: 5/34, p = 0.386), same colony foragers (treatment: 11/40, control: 5/34, p = 0.259) or foreign foragers (treatment: 35/40, control: 32/34, p = 0.442).

Aggression specific to newly emerged workers, as calculated by our index, was significantly affected by temperature (z = − 5.3, p < 0.001) but not by treatment (z = −1.0, p = 0.302).

## Discussion

The blank slate hypothesis to explain why newly emerged worker honey bees are accepted by any colony has not been sufficiently tested since its original formulation [18], perhaps because no viable alternative has been conceived to explain the overall absence of aggression towards newly emerged worker bees in general. The idea that the absence of cuticular hydrocarbons in newly emerged workers makes them unrecognizable has therefore persisted, which is difficult to test directly because the removal of hydrocarbons from live insects is problematic [23]. Here, we have proposed an alternative explanation for the near universal acceptance of young workers in honey bees, and social insects in general, by positing that adult workers are constantly exposed to newly emerged nestmates and learn this depauperate cuticular hydrocarbon profiles as belonging to their colony. Thus, they could learn the blank slate instead of failing to recognize it. Because newly emerged workers from other colonies are similarly devoid of cuticular hydrocarbons, they could be recognized as familiar and accepted as nestmates.

Learning influences nestmate recognition in many contexts [13–15], but we failed to find empirical support for this alternative hypothesis: Contrary to our prediction, honey bee workers that matured for three weeks without any contact to newly emerged bees were not significantly more aggressive towards newly emerged bees than workers that lived in normal colonies with a relatively steady emergence of, and thus exposure to, new workers. We found that aggression was not different between our experimental groups regardless of how we summarized aggressive behaviors or whether we adjusted for general aggressiveness, as measured by paired aggression assays of the same worker groups towards familiar or unfamiliar foragers. This may suggest that the absence of recognition cues is not learned in this context or does not modify subsequent behavior if learning does occur. Regardless, we conclude that regular exposure to newly emerged workers does not lead to a substantial behavioral difference towards newly emerged workers in an aggression assay. This provides some evidence against our hypothesis, although we cannot rule out that workers learn the depauperate cuticular hydrocarbon profile of newly emerged bees during their own emergence [20,24]. In turn, our study may be interpreted as indirect support for the original blank slate hypothesis [18], but the decisive test of whether honey bees can or cannot recognize the general absence of cuticular hydrocarbons remains to be performed.

As expected, the aggressive scores were highest in the assays performed with unfamiliar foragers from another colony than the experimental group. This outcome validates our behavioral test method, including the order of exposure to newly emerged, familiar foragers, and unfamiliar foragers. This order was chosen to minimize carry-over effects and indeed response scores were not correlated between the first and subsequent assays. It is important to note that much lower aggression was observed overall in several pilot tests of our assays that were carried out without the 24-hour acclimation period of the worker groups in the Petri-dish inside their own colony, which was the reason for our experimental timeline and highlights the context-dependency of tests for colony defense behavior.

Similar to the response towards newly emerged workers, aggression towards unfamiliar foragers was not affected by our experimental treatment. However, the experimental groups significantly differed in behavior towards foragers from their own colony, indicating that the emergence of new workers can affect the behavior of older workers towards nestmates. Workers that matured without exposure to newly emerged workers were more aggressive towards foragers from the same colony than workers that had newly emerging workers in their colony. We cannot exclude the possibility that the presence of newly emerging worker changes the cuticular hydrocarbons of older workers from the same colony that were introduced to the group in the assay. However, it seems more likely that the observed effect can be explained by indirect effects of newly emerged workers on the behavior of the group of bees that received the “intruder”. Our experimental treatment of removing ready-to-emerge brood increased the average worker age of these colonies compared to the controls. While the age composition is such an indirect effect [25], we would have predicted the opposite effect because the age-based behavioral maturation our focal cohort would have been slowed by an overall older colony worker population. Extensive aggression towards nestmates is not expected, although returning foragers are regularly inspected at their nest entrance and “false-positive” recognition errors occur [26]. Correspondingly, the higher aggression scores in the experimental group relative to the control group was largely due to more “antennation” and “crawl over” events and not “biting” and “stinging” behavior. Thus, the emergence of new workers in honey bee colonies decreases the likelihood of older workers to engage with foragers outside their natural context. Whether this finding is to be interpreted in the context of nestmate discrimination or has to do with the coordination of other colony functions remains to be elucidated by corresponding studies of entire colonies.

Our results also demonstrate an intriguing temperature dependency of the interactions of a group of worker bees towards different intruders: Aggression scores towards newly emerged bees decreased with temperature, while the same measures by the same workers towards nestmate foragers and unfamiliar foragers were not significantly affected by temperature, although positive trends existed. We note that these findings could be somewhat affected by the fact that different replicates were conducted at different average temperatures. A positive relation between temperature and aggressive behaviors could be explained by a simple increase in activity [21], which was our initial reason for including temperature in our analyses. However, behaviors that were high on our aggression scale increased more with increasing temperature than other behaviors, suggesting a potential specific effect on the responsiveness against foreign intruders. This effect could be explained by a higher volatility of any compounds that are used for nestmate discrimination in a warmer environment [27]. The response towards newly emerged workers was negatively affected by temperature, which could also be interpreted as support for temperature speeding up behavioral interactions, leading to nestmate acceptance in this case. Five extraordinarily high aggression scores were observed at relatively low temperature. In this case, the relatively low temperature may have led to initial recognition errors that were subsequently amplified by agonistic interactions and led to a rejection of those newly emerged bees from the group [28]. Overall, the contrasting findings on the temperature-dependency of our experiments suggests that the effect of temperature on nestmate recognition in honey bees is still incompletely understood.

## Supporting information

S1 Table All raw data plus calculated response indices from the study. (XLSX)
